# Integrins and Cell Metabolism: An Intimate Relationship Impacting Cancer

**DOI:** 10.3390/ijms18010189

**Published:** 2017-01-18

**Authors:** Rehman Ata, Costin N. Antonescu

**Affiliations:** 1 Department of Chemistry and Biology, Ryerson University, 350 Victoria Street, Toronto, ON M5B 2K3, Canada; rehman.ata@ryerson.ca; 2 Graduate Program in Molecular Science, Ryerson University, 350 Victoria Street, Toronto, ON M5B 2K3, Canada; 3 Keenan Research Centre for Biomedical Science of St. Michael’s Hospital, Toronto, ON M5B 2K3, Canada

**Keywords:** AMPK, mTOR, HIF1, membrane traffic, glycosylation, hypoxia, nutrient deficit, cancer metabolism, metabolic stress

## Abstract

Integrins are important regulators of cell survival, proliferation, adhesion and migration. Once activated, integrins establish a regulated link between the extracellular matrix and the cytoskeleton. Integrins have well-established functions in cancer, such as in controlling cell survival by engagement of many specific intracellular signaling pathways and in facilitating metastasis. Integrins and associated proteins are regulated by control of transcription, membrane traffic, and degradation, as well as by a number of post-translational modifications including glycosylation, allowing integrin function to be modulated to conform to various cellular needs and environmental conditions. In this review, we examine the control of integrin function by cell metabolism, and the impact of this regulation in cancer. Within this context, nutrient sufficiency or deprivation is sensed by a number of metabolic signaling pathways such as AMP-activated protein kinase (AMPK), mammalian target of rapamycin (mTOR) and hypoxia-inducible factor (HIF) 1, which collectively control integrin function by a number of mechanisms. Moreover, metabolic flux through specific pathways also controls integrins, such as by control of integrin glycosylation, thus impacting integrin-dependent cell adhesion and migration. Integrins also control various metabolic signals and pathways, establishing the reciprocity of this regulation. As cancer cells exhibit substantial changes in metabolism, such as a shift to aerobic glycolysis, enhanced glucose utilization and a heightened dependence on specific amino acids, the reciprocal regulation of integrins and metabolism may provide important clues for more effective treatment of various cancers.

## 1. Introduction

Integrins are a family of transmembrane proteins expressed in almost every cell type that mediate attachment to the extracellular matrix (ECM), and are critical regulators of cell physiology including cell migration and proliferation [1,2,3,4]. Dynamic membrane traffic (endocytosis and recycling) regulates many aspects of integrin function [5,6], including the formation of force-generating adhesions to the extracellular matrix and assembly of the actin cytoskeleton during cell migration [7].

Integrins are present on the cell surface as heterodimers consisting of an α and a β subunit [6]. In humans, there are 18 α-integrins and eight β-integrin subunits, which combine to form at least 25 αβ heterodimers [6]. In addition to establishing a physical bridge from the ECM to the actin cytoskeleton, integrins control the activation of a variety of intracellular signaling pathways, including the control of activation of actin nucleation, polymerization and cross-linking proteins, as well as pro-survival and mitogenic signaling [6]. Importantly, many of these signals can promote cancer cell growth and survival and thus contribute to cancer progression if the appropriate regulation is disrupted [5].

Integrins can exist on the cell surface in one of three conformations: inactive and bent with low affinity for ECM ligands, extended and primed with a closed head-piece and therefore low affinity for its ligand, or extended with an open head-piece with high affinity for extracellular matrix (ECM) ligands such as fibronectin, collagen, laminin and vitronectin [2]. The inactive integrin conformation is stabilized by a salt bridge between the α- and β-integrins in the cytoplasmic tail regions and helix packing in the transmembrane domain [8]. Generally speaking, integrin activation is regulated by two mechanisms: through the binding of proteins to the cytoplasmic tails, which induces conformational changes in the integrin heterodimer that facilitate interaction with ECM ligands, or through the engagement of extracellular matrix ligands on the exofacial portion, which induces integrin clustering and promotes activation [2,8].

Integrin heterodimers are the primary point of contact to the ECM in many cells [9]. Activation of integrins through engagement of ECM initiates with ligand binding and clustering (e.g., into focal contacts), which then facilitates the recruitment of proteins that stabilize activated integrins and establish a bridge to the cytoskeleton, including talin, vinculin, paxillin and α-actinin [7,10]. Some of these integrin clusters eventually mature from focal contacts to larger focal adhesions (FAs), which are important to provide traction forces required for migration [7]. The regulation of integrin conformation and thus affinity for ECM ligands can occur upon membrane recruitment and release of auto-inhibition of talin by binding to phosphatidylinositol-4,5-bisphosphate (abundant in the plasma membrane) or cleavage by calpain [3]. The subsequent binding of talin to β-integrins promotes integrin heterodimer activation, perhaps by relieving the inhibitory salt bridge between α and β integrins [11]. A complex network of protein interactions, with specificity for distinct integrin heterodimers, further regulates integrin activation, and which is described in several recent reviews [3,12].

### 1.1. Integrin Activation Elicits Proliferative and Survival Signaling

Although integrins themselves do not possess any kinase or other signaling activity, clustering and activation of integrins leads to recruitment and activation of a number of kinases and signaling adaptors, which allows integrins to serve as signaling centres that promote cell migration, cell survival and cell proliferation [3,13]. By this mechanism, integrins activate focal adhesion kinase (FAK) [14], integrin-linked kinase (ILK) [15], and Src-family kinases [16], as well as the signaling adaptor p130 CRK-associated substrate (p130CAS) [3,13]. These integrin-proximal signals can elicit activation of many canonical signaling pathways, including phosphatidylinositol-3-kinase (PI3K), leading to the production of phosphatidylinositol-3,4,5-trisphosphate (PIP3), and activation of Akt [17,18]. Other integrin-derived signals include the RAS- mitogen-activated protein kinase (MAPK) pathway [19], and Rho family GTPases [20]. Importantly, integrin signaling cooperates with that of growth factor receptors such as receptor tyrosine kinases, as reviewed by [20,21]. The regulation of integrins and growth factor receptors is reciprocal and complex, and can include regulation of gene expression, signal amplification by activation of common signaling intermediates, activation of one receptor by another, and in some instances physical association of integrins and growth factor receptors [22,23,24,25,26,27,28,29]. For example, β1 integrin silencing impairs normal activation of the epidermal growth factor (EGF) receptor (EGFR) upon binding EGF [25], and α5β1 integrin associates with EGFR and the related receptor ErbB3, thus enhancing activation of PI3K-Akt signaling [26]. Thus, while here we focus on the regulation and function of integrins, it is important to be mindful that integrins function as part of a broader signaling paradigm that exhibits reciprocal regulation with growth factor receptors such as EGFR.

In addition to the specific signaling of ECM ligand-bound, activated integrins, unliganded integrin complexes elicit apoptotic signals, linking detachment from the ECM to apoptosis, a phenomenon termed anoikis [30]. Disengagement of ECM by integrins triggers anoikis by removal of pro-survival signaling by FAK and other integrin-initiated signals, by disruption of focal adhesions leading to alterations in the actin cytoskeleton that impact mitochondrial targeting of apoptotic proteins, activation of pro-apoptotic signals such as p38 and c-Jun N-terminal kinase (JNK), and activation of the initiator caspase CASP8 [31]. Notably, specific integrins heterodimers vary in their ability to engage anoikis [31]. In general, signaling by activated, ligand-bound integrins serves to promote cell survival and proliferation, and controls adhesion and migration in coordination with growth factors and cytokines. 

### 1.2. Integrin Internalization and Membrane Traffic

Integrins can undergo internalization from the cell surface through clathrin-mediated endocytosis as well as clathrin-independent endocytosis [5,6,32,33]. While different integrin heterodimers may undergo distinct internalization, perhaps the most studied internalization mechanism is that of β1-integrin, which indeed exhibits context-dependent internalization. For instance, β1-integrin can be internalized via clathrin-mediated endocytosis [34,35,36,37] or clathrin-independent mechanisms [38]. For internalization via clathrin-mediated endocytosis, the β-integrin subunit contains a conserved NXXY motif on its cytoplasmic tail, which interacts with specific adaptor proteins (i.e., AP2, Dab, Numb) that recruit the receptor to clathrin endocytic structures at the plasma membrane [39]. For example, dab2 controls the clathrin-dependent internalization of α1β1, α2β1 and α3β1 (but not α5β1) integrins [36,37].

Once internalized, β1-integrin traffics to several distinct compartments during recycling, including to specialized Rab21 early endosomes, APPL1 early endosomes [40], Rab25 endosomes [41], and Rab4- and/or Rab11-recycling endosomes [5,6,42]. β1-integrin recycling is controlled (e.g., by growth factor stimulation) via regulation of Arf6, and Arf6 GAPs and GEFs such as ARNO, GRP1, ARAP2 and ACAP1 [40,43]. As part of the complex regulation of its membrane traffic, β1-integrin associates with Rab21 [44] and ACAP1 [45].

### 1.3. Integrins Control Cell Adhesion and Migration

As a result of interactions with the ECM, integrins have important roles to play in cell adhesion to and cell migration along specific substrata [46]. During cell migration, coordinated regulation of integrin membrane traffic and actin polymerization facilitate the formation of protrusions of filopodia and lamellipodia at the leading edge of a cell [7,47]. In general, disassembly of focal adhesions at the cell posterior, followed by internalization and recycling of integrins near the leading edge contributes to cell migration [48,49]. The interdependent formation of integrin-based nascent adhesions and focal complexes and dynamic actin polymerization within the lamellipodium of a migrating cell allows for traction generation for cell migration [7]. At the cell posterior, a coordinated release of integrins is partly due to contractile forces, which severs the connection of integrins with the actin cytoskeleton, either leaving the integrin bound to the substratum as integrin footprints [50,51] or triggering integrin endocytosis [6].

### 1.4. Integrins and Cancer

Given that integrins control pro-survival and proliferative signaling as well as cell migration, integrins have important functions in cancer growth and metastasis. Integrins are not themselves oncogenes, but integrin functions support many other alterations in cancer [4]. Many solid tumours are of epithelial origin, and retain some expression of epithelial integrins, including α6β4, α6β1, αvβ5, α2β1 and α3β1, but the expression of some of these is altered in some tumours, and some tumours exhibit high levels of additional integrins such as αvβ3, α5β1 and αvβ6 [4]. While many integrins elicit pro-survival and proliferative signals, certain specific integrins, such as α5β1 may elicit negative regulation of these processes [52,53], whereas αvβ3 elicits positive [54] or negative [55] signals for survival depending on cell context. Further, alterations in integrin expression profile can protect cells from anoikis [30], as evinced by the example of anoikis avoidance resulting from switching expression of αvβ5 to αvβ6 integrins in squamous cell carcinomas [56].

Alterations in the normal membrane traffic of integrins also contribute to cancer phenotypes, in particular to enhanced invasive migration. Certain tumours express Rab25, which interacts with α5β1 integrin and promotes invasive cell migration [57] as a result of unique non-degradative membrane traffic through the late endosome/lysosome [41]. Moreover, Rab13 expression is elevated in some invasive cancers, and Rab13 promotes recycling of integrins and other proteins to the leading edge to enhance migration [58].

Integrins are key regulators of epithelial-mesenchymal transition (EMT), a phenomenon that increases cancer cell motility and invasiveness [59]. The complex reprogramming of gene expression required for down-regulation of epithelial-specific genes and up-regulation of mesenchymal phenotype genes requires integrin signaling, as evinced by the requirement for α3β1 integrin for TGFβ1-stimulated Smad signaling to promote EMT [60]. EMT also requires changes in cell adhesion and expression of specific integrins, such as down-regulation of β4 integrin upon stimulation of epithelial cells with TGFβ1 during EMT [61]. Specific integrins also control the activity and localization of matrix metalloproteases to facilitate invasive migration, such as the control of matrix metalloproteases 9 (MMP9) by αvβ3 integrin in MDA-MB-435 breast cancer cells [62]. Modulation of integrin function also contributes to angiogenesis, and controls the contribution of stromal cells within the tumour microenvironment to cancer cell growth [4,63].

Demonstrating the key roles played by specific integrins in various cancers, the expression of specific integrins in certain cancers can be correlated with cancer outcome [4,64]. In general, the alterations of integrin expression, activation, membrane traffic and signaling are diverse and effect context-specific regulation of tumour growth, survival and migration. Nonetheless, insight into the mechanisms that underlie the changes in expression and function of integrins during cancer progression are critical to understanding how integrins control cancer.

Collectively, these studies indicate that alterations in the expression profile of specific integrins, integrin activation, integrin signaling to control proliferation and survival, as well as integrin membrane traffic are phenomena that underlie the growth and survival of many tumours. Understanding how specific hallmarks of cancer establish control of these properties and functions of integrins is important to better understand how integrins contribute to tumour growth and to develop new therapies to target cancer. One of the key hallmarks of cancer is alteration in cell metabolism, with it recently re-emerging at the forefront of cancer biology after some of the initial work describing altered cancer cell metabolism by Otto Warburg [65]. Here, we examine the reciprocal regulation of integrins and cell metabolism, in the context of interdependent alterations of integrin function and altered cell metabolism in cancer cells.

## 2. Metabolic Signals and Alterations in Cancer

Cells must coordinate a number of their processes and activities with their metabolism, as the latter provides energetic and biosynthetic considerations for every aspect of cell physiology. Nearly every human cell can experience metabolic stress (e.g., low cellular (ATP)) as a result of hypoxia, ischemia, fluctuations in the availability of specific nutrients, increased metabolic demand or production of reactive oxygen species (ROS) [66]. A number of cellular metabolic sensor systems respond to metabolic stress or sufficiency and function to elicit adaptive responses to ensure cell survival and homeostasis. These include AMP-activated protein kinase (AMPK), mammalian target of rapamycin (mTOR) and hypoxia-inducible factor (HIF), which collectively integrate signals of nutrient scarcity or availability and environmental conditions to coordinate cellular homeostasis during metabolic stress.

Cancer cells have unique metabolic considerations, and thus exhibit distinct control of signaling proteins that sense and indicate metabolic scarcity or availability. In general terms, as cancer cells exhibit a substantially higher rate of proliferation than cells from healthy adult tissues, they also exhibit a higher demand for metabolic intermediates (e.g., nucleotides, phospholipids, certain amino acids) and thus on the biosynthetic pathways that are responsible for uptake or production of these intermediates [67,68]. Many cancer cells exhibit a shift in glucose metabolism under aerobic conditions from oxidative phosphorylation (which efficiently produces ATP) to aerobic glycolysis, where metabolic intermediates from glycolysis are rerouted to pathways for production of other biosynthetic precursors, such as serine biosynthesis. Indeed, this is often accompanied by a higher demand for glucose entry into glycolysis, which results in a long-appreciated elevated glucose uptake into cancer cells [69].

Several mechanisms have been demonstrated for alterations to glucose metabolism in cancer cells to favour generation of metabolic intermediates. For example, many cancer cells express the M2 isoform of pyruvate kinase (PK) instead of the M1 isoform that is ubiquitous in adult tissues [70,71]. The M2 but not the M1 isoform of PK promotes tumour cell growth [71], due to PKM2 being sensitive to inhibition by mitogenic and proliferative signaling pathways [72], conditions which enhance lactate production or shuttling of intermediates to serine biosynthesis pathways, instead of directing metabolites for entry into the Kreb’s cycle for ATP production. In turn, serine, glycine and a number of other nutrients (either the products of biosynthesis or uptake from the extracellular milieu) provide substrates for entry into a number of metabolic cycles (e.g., folate and methionine), which in turn contribute to the synthesis of nucleosides and phospholipid headgroups, protein translation, and a number of other key metabolic functions [73]. Indeed, systematic metabolic profiling of the NCI-60 cancer cell panel identified an elevated rate of glycine metabolism in highly proliferative cells, while perturbation of glycine availability selectively impacted highly proliferating cells [74]; other studies also support a key requirement for glycine or serine for cancer cell proliferation [75,76,77]. A high rate of glutamine metabolism in tumours that exceeds the requirement for protein and nucleotide synthesis further facilitates the production of biosynthetic precursor molecules during glucose metabolism by providing reductive capabilities in the form of NADPH [78]. Fatty acid oxidation provides additional ATP production capabilities to tumours as required under some circumstances [79]. The transfer of palmitate into the mitochondria represents the rate-limiting step for fatty acid oxidation [80]. Indeed, a specific isoform of carnitine palmitoyltransferase (CPT1C) is frequently upregulated in human lung tumours, and perturbation of CPT1C reduced the growth of tumour xenografts and rendered cells more sensitive to metabolic stress [81]. These studies suggest that fatty acid oxidation may critically contribute to ATP production under some conditions of metabolic insufficiency in tumour cells. We direct the reader to recent comprehensive reviews for further reading on the metabolic alterations in cancer cells [67,68,73,82,83,84,85,86].

In addition to these largely cell-autonomous considerations for cancer cell metabolism, the tumour microenvironment also imposes on cancer cells specific metabolic constraints [87]. The reduced blood flow and high interstitial pressure of some tumours can result in a tumour microenvironment that is hypoxic, that has scarcity of specific nutrient(s) and/or is impacted by specific consequences of tumour metabolism [88]. An example of the latter is the altered pH of the tumour microenvironment [89,90], which can result from high rates of lactate production and extrusion [91]. This elevated lactate production results from metabolic reprogramming of tumours and may also reflect the hypoxic or nutrient-constrained tumour microenvironment that has complex effects on specific tumours [91,92]. Thus, the tumour microenvironment imposes metabolic constraints, including metabolic insufficiency, to further the unique metabolic profile of cancer cells relative to healthy tissues [88].

Collectively, from this work emerges the notion that cancer cells exhibit distinct requirements for specific metabolites, while favouring the formation of specific biosynthetic precursors over the high rate of ATP production in the mitochondria. As such, cancer cells may have distinct activation of specific sensors of energy sufficiency or stress, which we discuss next.

### 2.1. Metabolic Control of AMP-Activated Protein Kinase (AMPK) and Its Role in Cancer

AMPK is engaged during energy insufficiency, as it becomes activated upon an increase in the AMP:ATP (or ADP:ATP) ratio [93]. This kinase is a heterotrimer and AMP, ADP, and ATP directly bind to the γ subunit, resulting in control of the serine/threonine kinase activity of the α-subunit [93]. AMPK is activated [94] when T172 of the α-subunit is phosphorylated [93]. This T172 phosphorylation is mediated by either the LKB1-STRAD-MO25 complex [95,96,97] or the calcium/calmodulin-activated protein kinase kinases (CAMKKβ) [98,99,100]. AMP but not ATP binding impairs dephosphorylation [101,102], such that AMPK phosphorylation and thus activity is enhanced by a reduction in ATP levels relative to AMP and ADP. AMPK activity can also be regulated independently of AMP:ATP, including by reactive oxygen species (ROS) [103] and nitric oxide (NO) [104]. Further, AMPK activity is also regulated by hormones that control systemic metabolism such as adiponectin [105,106], leptin [107], thyroid hormone [108,109], ghrelin [110], and cannabinoids [111]. AMPK is also activated by a number of pharmacological agents, including the anti-diabetic agent metformin [112].

Upon activation, AMPK leads to enhancement of nutrient uptake and energy production, and energy conservation, through phosphorylation of a number of substrates [93,113]. Many lines of evidence suggest that AMPK is an important regulator of cancer growth and proliferation, which has been recently extensively reviewed [114,115,116]. The AMPK activator LKB1 is a potent tumour suppressor [117]. Furthermore, AMPK directly phosphorylates and controls p53 in order to affect cell cycle arrest [118], and AMPK negatively regulates anabolic pathways required for cancer growth, including fatty acid and protein synthesis [93], in part by direct phosphorylation and activation of TSC2 by AMPK, resulting in impairment of mTOR signaling [119]. These and other studies indicate that AMPK activation serves to limit cancer cell growth and survival.

In contrast, under some circumstances, AMPK activation by nutrient deficit and metabolic stress may promote tumour survival, by enhancing NAPDH levels via suppression of fatty acid synthesis and enhancement of fatty acid oxidation [120], the latter which may result from AMPK-dependent upregulation of CPT1C [81], and by activating the p38-PGC1 transcriptional axis [121]. Thus, under different contexts, AMPK activation in tumours controls cohorts of cellular functions that can result in either enhancement or impairment of cell viability or proliferation, which may reflect selective and district functions of AMPK in early versus late stages of cancer progression [115]. Nonetheless, these studies collectively indicate that the altered cell autonomous and microenvironment-imposed metabolism of tumours often triggers AMPK activation within tumour cells.

### 2.2. Mammalian Target of Rapamycin (mTOR) Integrates Amino Acid Sensing and Mitogenic Signaling

mTOR is part of two distinct complexes, mTORC1 and mTORC2, which differ in binding interactions with components of each complex, in mechanisms of regulation and in substrate specificity [122]. While both complexes contain mTOR, Deptor and mLST8, the mTORC1 complex is also comprised of Raptor, and PRAS40, whereas the mTORC2 complex is also comprised of Rictor, Proctor and mSIN1 [123]. The nature of regulatory inputs into mTORC1 make it a key integrator of metabolic and mitogenic cues [122], and as such we focus here on mTORC1.

mTORC1 is activated by signaling by growth factors or ECM engagement by integrins as a result of activation of phosphatidylinositol-3-kinase (PI3K), leading to the production of phosphatidylinositol-3,4,5-trisphosphate (PIP3), which in turn activates Akt, a serine/threonine kinase. Akt phosphorylates TSC2, a GAP protein that is part of the Tuberous Sclerosis Complex (TSC), resulting in reduced GAP activity towards the GTPase RAS homologue enriched in brain (RHEB) [124], which in turn controls mTORC1 activity. Overall, this PI3K-Akt pathway results in activation of mTORC1 upon stimulation with growth factors.

In contrast to activation by growth factor signaling, mTORC1 regulation by amino acids is independent of TSC. The Rag family of GTPases are critical for amino acid-induced activation of mTORC1 [125,126]. The Ragulator complex interacts with Rags and recruits mTORC1 to the surface of lysosomes in the presence of amino acids [127]. This recruitment of mTORC1 to lysosomes is required for activation of this kinase by amino acids [127], as is sensing of amino acids within the lysosome lumen by the V-ATPase [128]. This lysosomal sensing system for amino acid availability functions in conjunction with various amino acid transporters localized to the plasma membrane and to the limiting membrane of the lysosome [129], such as LAT1-4F2hc for the transport of Leucine [130], ensuring that mTORC1 can be activated by amino acid availability resulting from lysosomal degradation or from other sources (e.g., uptake from the extracellular milieu or biosynthesis). Rag-independent activation of mTORC1 [131,132,133], such as by glutamine [132,133], further expands the metabolic signals that control mTORC1.

Many cancer cells have enhanced mTORC1 activity resulting from upregulation of PI3K-Akt signaling, either due to enhanced mitogenic receptor activity, inactivating mutations in the negative regulator of PI3K signaling, phosphatase and tensin homolog (PTEN), or activating mutations in PI3K or Akt, or other mechanisms [122]. Activated mTORC1 enhances protein translation by phosphorylation of p70S6K, which in turn phosphorylates the 40S ribosomal protein S6, and by phosphorylation of 4EBP1, which suppresses inhibition of eIF4E; each of these processes enhances translation of specific transcripts [134]. mTORC1 also promotes lipid synthesis by control of Lipin-1 to promote sterol regulatory element-binding protein (SREBP)-dependent transcription [135], and negatively regulates autophagy, such as by phosphorylation of ULK1 [136]. mTORC1 also control glycolytic flux to ensure suitable ATP and biomass production [137]. These examples illustrate a broader function of mTORC1 to enhance anabolic processes that are critical for many of the hallmarks of cancer, and demonstrate the contribution of active mTORC1 within cancer cells.

### 2.3. Hypoxia Sensing by HIF1 Coordinates Metabolic Adaptation

The hypoxic microenvironment of many tumours is sensed by a machinery that leads to activation of HIF1α, a transcription factor, as reviewed by [138,139,140]. HIF1α increases the transcription of many genes such as the facilitative glucose transporter GLUT1 and glycolytic enzymes such as phosphofructokinase (PFK) and many others [141]. HIF1α also impairs mitochondrial metabolism of glucose by a number of mechanisms, such as by limiting pyruvate produced by glycolysis from entering the Kreb’s cycle, as a result of HIF1α-dependent inactivation of pyruvate dehydrogenase resulting from increased expression of pyruvate dehydrogenase kinase [142]. Collectively, the profile of genes and processes induced by activated HIF1α is consistent with genes required to establish the Warburg effect.

HIF1α acts as a sensor of O_2_ levels due to hydroxylation on P402 and P564 by prolyl hydroxylases during normoxic conditions [143,144,145]. Hydroxylated HIF1α is recognized by von Hippel Landau protein (VHL), which mediates ubiquitinylation and degradation of HIF1α [146,147]. Under hypoxic conditions that limit HIF1α hydroxylation, this transcription factor is stabilized and functional [148]. In addition to activation under hypoxic conditions, HIF1α can also be stabilized under a number of normoxic conditions, including as a result of activation of PI3K-Akt-mTOR signaling [149], Ras [150] and Src [151], each of which can be upregulated in certain cancers.

As integrins play a central role in the control of cell physiology, such as by controlling proliferative and pro-survival signaling and by directing cell adhesion and migration, integrin function must be highly regulated and coordinated with metabolic cues. Indeed, the integration of metabolic signaling into the regulation of a variety of distinct cellular processes has emerged as a central paradigm of cell physiology, and metabolic heterogeneity may underlie many context-dependent cell behaviours. Here, we examine the interdependent and reciprocal regulation of integrins and cell metabolism in the context of cancer cell proliferation, survival, adhesion and migration. We examine the regulation of integrin expression and function by metabolic signals and cues, the evidence for control of nutrient uptake and cellular metabolic pathways by integrins, and how this interdependent regulatory relationship may underlie some of the hallmarks of cancer.

## 3. Regulation of Integrins by Metabolic Cues and Signaling

**Figure 1:**
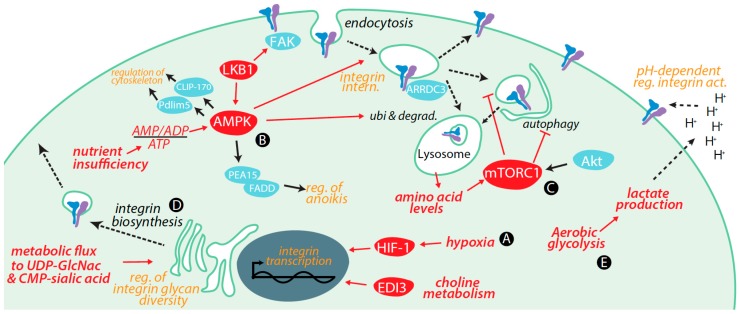
Regulation of integrins by metabolic cues and signaling. (**A**) Transcriptional regulation of integrins by hypoxia increases expression of α5, β1, and β2 integrins via hypoxia inducible factor (HIF)-1; (**B**) AMP-activated protein kinase (AMPK) activation elicits internalization of cell surface β1 integrin without affecting expression levels. Long term AMPK activation (24 h) with berberine induced β1 integrin degradation and impaired cell migration. Further control by ubiquitinylation and degradation of β4 integrin occurs via the α-arrestin protein Arrestin Domain Containing 3 (ARRDC3). Cell migration is further controlled by AMPK phosphorylation of Pdlim5 and CLIP-170 to regulate cytoskeleton dynamics. AMPK activation prevents anoikis by phosphorylating phosphoprotein enriched in astrocytes 15 kDa (PEA15), which can then bind to Fas-Associated protein with Death Domain (FADD) to prevent recruitment of initiator caspases; (**C**) The Arf4-dependent internalization and recycling of α5β1 integrin is regulated by mTORC1, a sensor of amino acid levels. mTORC1 and AMPK have opposite effects on ULK1-dependent autophagy, thus exerting control of integrins by control of autophagy; (**D**) Glucose and glutamine metabolism allows generation of UDP-GlcNAc, which together with metabolic production of CMP-sialic acid controls the glycan profile of integrins and thus integrin function; (**E**) Aerobic glycolysis, which is commonly observed in tumours, can lead to reduced extracellular pH. Alterations in extracellular pH control integrin structure, and integrin-dependent cell adhesion and migration. Red lines indicated positive regulation (arrowheads) or negative regulation (bars).

Metabolic signals and cues and key metabolites exert control over integrins by several mechanisms, including by regulation of the following: transcription and degradation of integrins, integrin membrane traffic, integrin glycosylation (a key post-translational modification), integrin signaling, and the tumour microenvironment (such as by control of extracellular pH) (Figure 1). In this section, we focus on control of integrin function by key metabolic signals in cancer cells, and highlight metabolic control of integrin function in non-cancer cells in pertinent circumstances.

### 3.1. Transcriptional Control of Integrin Expression by Metabolic Signals

Several examples have been reported of the control of transcription of specific integrins by metabolic cues, which may underlie the altered expression of integrins in cancer. Through HIF1-dependent mechanisms, hypoxia induces transcription of α5 integrin in SW480 human colon cancer cells [152], of β1 integrin in 18CO colon fibroblasts [153], of α5β1 integrin in osteosarcoma cells [154], and of β2 integrin in U937 leukocytes [155]. Further, stimulation of prostate cancer cells with adiponectin results in increased transwell migration, and increased transcription and expression of α5β1 integrin, in an AMPK-dependent manner [156]. Adiponectin also induces expression of α2β1 integrin and enhanced migration in SW1353 and JJ012 chondrosarcoma cells in an AMPK-dependent manner [157].

EDI3, an enzyme functioning in choline metabolism, also regulates cell migration [158,159]. High expression of EDI3 increases the risk of metastasis in ovarian and endometrial cancers. Gene array analysis of MCF-7 breast cancer cells revealed that EDI3 controlled transcription and expression of β1-integrin and many other integrin-related signaling genes [160]. Indeed, silencing of EDI3 reduced cell spreading, decreased cell attachment, and delayed protrusion formation [160], highlighting that the link between choline metabolism and integrin expression controls cell adhesion and migration. 

The integrin heterodimer αVβ3 is overexpressed in ovarian cancers and has been linked to thyroid hormone signaling, a key systemic metabolic regulator. The unique binding of αVβ3 integrin to the thyroid hormones 3,4,5′-triiodo-l-thyronine (T3) and l-thyroxine (T4) [161], but not engagement of arginyl-glycyl-aspartic acid (RGD) motifs, increases transcription of αv and β3 integrin genes in an MAPK-dependent manner [161]. This unique regulation of αVβ3 by thyroid hormones illustrates control of integrin transcription by systemic metabolic cues.

### 3.2. Control of Integrin Membrane Traffic by Metabolic Signals

Using a mass spectrometry-based approach, we identified that AMPK elicits broad control of the membrane traffic of cell surface proteins, such that AMPK activation redistributes a large cohort of plasma membrane proteins to intracellular compartments [162]. GO classification of proteins revealed that cell adhesion and migration proteins, including α4 and α11 integrins, had reduced cell surface abundance upon AMPK activation. Using other methods, we also confirmed that AMPK activation resulted in a reduction in cell surface levels of β1-integrin, but not total β1-integrin expression, which correlated with impaired cell migration [162].

Studies of cells lacking expression of ARNT1 and HIF1α, two components of the HIF1 trimeric complex, revealed that HIF1 controls cell surface levels of αvβ3, but not that of other integrins (e.g., β1 and β5 integrins), and that this regulation of integrins did not involve control of integrin transcription and translation [163]. Instead, HIF1-deficient cells exhibited alterations of localization of αvβ3 integrin with Golgi markers, suggesting that HIF1 directs specific intracellular membrane traffic processes that selectively control the cell surface levels of αvβ3 integrin.

Furthering the understanding of the control of integrin membrane traffic by metabolic signals, the internalization and recycling of α5β1 integrin is regulated by amino acid availability, in an mTORC1-dependent manner [164]. The Arf4-dependent internalization of α5β1 integrin is also required for mTORC1 lysosomal recruitment and activation, demonstrating the reciprocal regulation of integrin membrane traffic and mTORC1 activation [164]. While some of the mechanisms remain to be elucidated (e.g., the mechanism by which AMPK controls integrin internalization), these findings illustrate that AMPK and mTORC1 metabolic cues may control integrins by direct control of integrin cell surface abundance and membrane traffic, thus impacting integrin-dependent cell migration.

### 3.3. Control of Integrin Degradation by Metabolic Cues

Prolonged cell stress or nutrient deprivation can induce autophagy, a mechanism involving the formation of a double membrane compartment within the cytosol termed an autophagosome, encapsulation of certain proteins and/or organelles within this compartment, followed by fusion with the lysosome for degradation [165,166,167]. By this process, autophagy releases biochemical intermediates, allowing cells to survive periods of nutrient deficit. The induction of autophagy is controlled by multiple inputs: it is inhibited by mTORC1 through the phosphorylation of ULK1, and is induced by AMPK, as a result of AMPK-dependent activation of TSC2 or direct phosphorylation of ULK1 at a site different than that phosphorylated by mTORC1 [168]. Induction of autophagy elicited an enhancement of β1-integrin recruitment to autophagosomes, leading to β1-integrin localization to lysosomes and β1-integrin degradation [168]. Autophagy induction may limit β1-integrin recycling following internalization, and thus favour β1-integrin degradation. In contrast, hypoxia promotes recycling of α6β4 (but not α3β1) integrin through control of Rab11-dependent recycling and microtubules in MDA-MB-231 breast cancer cells [169].

Consistent with metabolic cues impacting integrin function by regulation of integrin degradation, prolonged (24 h) treatment of SW480 and HCT116 colon cancer cells with the plant-derived benzylisoquinoline alkaloid berberine resulted in AMPK activation, and AMPK-dependent impairment of cell migration and degradation of β1-integrin along with attenuation of integrin signals (e.g., FAK phosphorylation) [170]. Further, the internalization, ubiquitinylation and degradation of β4 integrin is controlled by an α-arrestin protein, ARRDC3, as a result of control of binding of ARRDC3 to a phosphorylated form of β4 integrin [171]. Importantly, this control of β4-integrin by ARRDC3 limited breast cancer cell growth, migration (measured by wound assay), invasiveness (Matrigel assay) and anchorage-independent growth in MDA-MB-231 breast cancer cells [171]. ARRDC3 has an important role in controlling systemic energy utilization, by regulation of β1-adrenergic signaling in adipocytes [172]. The α-arrestin family to which ARRDC3 belongs has 14 members [173], some of which function as endocytosis adaptor proteins at the cell surface, and have emerging roles in mediating control of cellular processes by metabolic cues and reciprocally controlling cell metabolism [174]. Thus, α-arrestins such as ARRDC3 may function to bridge cellular and systemtic metabolic cues to control integrin membrane traffic and function.

### 3.4. Further Control of Integrin Expression by Metabolic Signals

In addition to the studies noted which have reported the control of integrin expression by metabolic cues through control of transcription or degradation, other studies have also reported control of integrin expression by specific metabolic cues. Overexpression of Akt2 in several breast and ovarian cancer cells leads to increased β1-integrin expression, invasion and metastasis on collagen IV substrates [175]. While Akt signaling could regulate β1-integrin expression by a number of mechanisms, one of the major outcomes of Akt signaling is activation of mTORC1. In addition, incubation of glomerular epithelial cells with high glucose concentration (25 mM) causes changes in expression of a number of integrins, resulting in decreased expression of integrins α2, α3, and β1 and increased expression of α5, αv, and β3 integrins [176]. In addition, human proximal tubular epithelial cells (HK-2) treated with high glucose (25 mM) exhibited reduced expression of α3, β1, αvβ3, and α5 integrins, and an increase of α2 integrin, as well as an increased adhesion to collagen IV or laminin [177]. These studies complement others that found that metabolic cues and signals control integrin expression by control of transcription or degradation.

### 3.5. Integrin Glycosylation Is Controlled by Metabolic Inputs 

Virtually all cell surface proteins, including integrins, are *N*-glycosylated, a post-translational modification that occurs during biosynthesis beginning in the endoplasmic reticulum and continuing with glycan processing in the Golgi. Many different integrin glycoforms have been reported in different cell types and physiological contexts, in particular for β1-integrin, reviewed by [178,179,180,181]. Importantly, the two most variable properties of integrin glycans are sialylation and β1-6 branching [178], and both of these molecular variations are impacted by metabolic cues and signals and have consequences for cancer cell growth, survival and/or migration, as we discuss below.

The Golgi-localized β1-6 *N*-acetylglucosaminyltransferase V GnT-V (also known as Mgat5) generates β1-6 branched glycans, which can be further modified by additional glycosyltransferases. Importantly, many tumours exhibit upregulation of GnT-V, in part due to the increased GnT-V expression due to signaling by a number of oncogenes. Many studies support a role for GnT-V-dependent β1-6 glycan branching in tumour progression or metastasis [178]. For example, increased expression of GnT-V in human fibrosarcoma HT1080 cells elicits selective increase in β1-integrin (but not α5-integrin) β1-6 glycan branching, which reduced cell adhesion and spreading on fibronectin, thus impacting tumour migration [182]. Importantly, the β-1,6-*N*-acetylglucosamine branched glycans produced by GnT-V are high affinity ligands for binding by galectin-3, and this interaction leads to the control of the clustering, signaling and membrane traffic of certain proteins within a galectin lattice at the cell surface [183]. Indeed, the GnT-V-dependent control of integrin glycan profiles allows galectin-3-dependent activation of FAK and PI3K, and impacts cell motility [184]. Galectin-3 facilitates cancer growth and metastasis by several mechanisms [185,186], further highlighting the importance of the GnT-V and β-1,6-*N*-acetylglucosamine branched glycans in cancer.

Notably, the reaction catalyzed by GnT-V is not only controlled by expression of this glycosyltransferase, but is also under metabolic control, as a result of metabolic flux through the glycosamine pathway that generates UDP-*N*-acetylglucosamine (UDP-GlcNAc), a key substrate for GnT-V-dependent β1-6 glycan branching [187,188]. Indeed, β1-6 glycan branching is sensitive to UDP-GlcNAc concentration, producing a switch-like response in glycan branching upon increasing UDP-GlcNAc availability [188]. This phenomenon places integrin-dependent adhesion, migration, and signaling under the control of glucosamine metabolism, which is in turn sensitive to glucose and glutamine metabolism [189]. Consistent with this interpretation, mutational inactivation of mitochondrial oxidative phosphorylation resulted in elevated glucose and reduced oxygen consumption, an elevation of β1-6 branching of *N*-glycans on β1-integrin, and increased cell motility and migration, suggesting that mitochondrial signals or glucose metabolic flux controls integrin function via control of glycan diversity [190].

The sialylation of N-linked glycans occurs by the action of sialyltransferases, in particular β-galactoside α2,6-sialyltransferase I (STGal6 I), and often occurs on β1-6 branched glycans [178]. Increased expression of STGal6 I and increased sialylation of various proteins, including integrins, has been well documented to promote tumour malignancy [178,191,192,193]. The glycosylation profile of α3β1 integrin in metastatic A375 human melanoma cells exhibits sialylated tetra-antennary oligosaccharides, and α3 integrin with β1-6 branched structures [194]. Importantly, enzymatic removal of sialic acid increased adhesion and impaired invasiveness, suggesting that regulation of integrin sialylation controls integrin-dependent cell functions. Consistent with this, ST6Gal I expression was required for β1-integrin sialylation and enhanced adhesion to and migration along collagen substrates in colon adenocarcinoma cells [195], and ST6Gal-I-dependent α2-6 sialylation of integrins in mouse hepatocarcinoma H22 cells increased α5β1 integrin-dependent cell adhesion to fibronectin [196]. In contrast, other studies reported that sialylation of α2β1 and α5β1 integrins impaired adhesion on collagen IV in MDA-MB-231 cells [197], indicating that while sialylation robustly regulates integrin-mediated cell adhesion and migration, the nature of this regulation may be context-specific for specific integrin heterodimers and specific ECM substrate combinations.

Importantly, like the formation of β1-6 branched glycans, sialylation is under metabolic control, as incorporation of sialic acid into *N*-glycan structures is sensitive to the presence of specific sugars in the culture media of CHO cells [198]. Integrins (esp. α6 integrin) are some of the selective *N*-glycoproteins that undergo increased sialylation as a result of increasing metabolic flux through the sialic acid pathway. The latter was demonstrated by treatment of SW1990 pancreatic cancer cells with the substrate 1,3,4-*O*-Bu3ManNAc, which led to increased metabolic flux to CMP-sialic acid, which in turn resulted in increased integrin sialylation [199]. Moreover, in U-87 MG glioblastoma multiforme cells, hypoxia altered expression of many genes, including an upregulation of ST3 β-galactoside α-2,3-sialyltransferase 6 (ST3Gal6) [200]. Interestingly, RAW264.7 cultured under hypoxic conditions or treated with CoCl_2_ to mimic hypoxia exhibited a robust increase in CMP-sialic acid, a precursor for *N*-glycan sialylation [189]. Taken together, these studies suggest that sugar nutrient and oxygen availability can exert substantial control over protein sialylation, and that regulation of sialylation of integrins in this manner may link tumour microenvironment metabolic cues with integrin function in tumour cells.

In addition to N-linked glycosylation that occurs during initial protein biosynthesis on exofacial protein domains, the dynamic post-translational modification of endofacial protein domains with *O*-linked β-*N*-acetylglucosamine (O-GlcNAc) also regulates a number of activities of various proteins. The O-GlcNAc modification of specific proteins involves O-GlcNAc transferase (OGT), and O-GlcNAc is removed by the action of O-GlcNAcase. O-GlcNAc interfaces with protein phosphorylation, and specific O-GlcNAc modifications can enhance while others impair phosphorylation on specific proteins [201]. Modification of proteins by O-GlcNAc requires UDP-GlcNAc as a substrate, indicating that like the activity of GnT-V, O-GlcNAc modification of specific proteins is sensitive to nutrient supply by specific metabolic pathways [202]. As such, the O-GlcNAc modification of specific proteins may function as a metabolic control to “calm” protein phosphorylation networks by linking these to metabolic state, as occurs for Akt-mTOR signaling [202]. In addition to direct sensing of nutrient availability, OGT expression is also enhanced by AMPK activation [203]. Indeed, certain integrins may be regulated by direct interplay between phosphorylation and O-GlcNAc modification, as has been proposed for β3-integrin [204], yet how O-GlcNAc modification of integrins or integrin signaling proteins may control integrin function remains to be more broadly addressed. Collectively, these studies have revealed the widespread control of integrin function by specific glycan modifications. The control of integrin glycan diversity by direct translation of nutrient and metabolic flux into regulation of glycan prolife or by activation of signals like AMPK and HIF further demonstrate the metabolic control of integrin-dependent functions.

### 3.6. Metabolic Signals Control Cell Migration and Adhesion

The control of integrin expression, membrane traffic, post-translational modification and signaling may be part of a broader control of cell adhesion and migration proteins by metabolic cues. Indeed, we found that AMPK activation controls the cell surface abundance of a number of cell adhesion and migration proteins, including a number of α integrins, and cadherin family proteins [162]. Importantly, AMPK also controls cell migration by direct phosphorylation of CLIP-170, a capping protein that controls microtubule dynamics important for cell migration [205], and also by control of actin cytoskeleton dynamics in the lamellipodium, through phosphorylation of Pdlim5 [206]. AMPK activation also impairs breast cancer cell migration upon stimulation with adiponectin [207]. Moreover, in endothelial progenitor cells, hypoxia induces AMPK activation and decreases β1- and α5-dependent adhesion on fibronectin, suggesting that AMPK controls β1 and α5 integrins [208]. Interestingly, silencing of the facilitative glucose transporter GLUT1 in MDA-MB-231 or Hs578T breast cancer cells resulted in decreased expression of β1 integrin, reduced Src and FAK phosphorylation, and decreased cell growth and migration [209]. While GLUT1 silencing also reduced expression of and signaling by EGFR, suggesting broad control of cell functions by glucose uptake and metabolism, this work is consistent with control of integrins and cell migration by metabolism and metabolic stress.

### 3.7. Metabolic Cues Control Integrin Signaling

Several studies have also reported control of integrin activation by metabolic cues, a phenomenon that could be related to the control of integrin expression, membrane traffic and post-translational modification described above. Some metabolic signals also control integrin signaling by controlling the expression of specific genes involved in signaling. Treatment of nasopharyngeal carcinoma cells with the AMPK activator metformin, along with rosiglitazone (an activator of PPARγ transcription factor) results in an AMPK-dependent decrease in ILK expression [210] and impaired growth of these cells. Consistent with this, treatment of non-small cell lung cancer cells with emodin resulted in AMPK activation and AMPK-dependent reduction of ILK expression, again impairing cell growth [211].

Metabolic signals can also control integrin signaling by other mechanisms. Treatment of smooth muscle cells with CoCl_2_ to mimic hypoxia resulted in HIF1α-dependent reduction in cell migration and adhesion, and also triggered a loss of FAK phosphorylation without changing FAK expression [212]. The AMPK-activating kinase LKB1 associates with and represses activation of FAK to control focal adhesion dynamics and cell migration direction persistence, although whether this may act through a metabolically regulated intermediate such as AMPK remains to be determined [213]. AMPK also negatively regulates signaling from Akt to mTORC1 [119], and as such down-regulates PI3K signaling downstream of integrin activation.

Consistent with these studies, an siRNA gene silencing screen performed in PC3 prostate cancer cells revealed several novel genes that control β1-integrin activation, including AMPK [214]. While this study did not resolve the mechanism by which AMPK controls integrin activation, this may involve control of expression of integrin signaling proteins, and/or may be related to the internalization of β1-integrin observed upon AMPK activation [162].

As discussed above, one of the key phenomena controlled by integrin-derived signals is anoikis, and metabolic signals also control this aspect of integrin signaling. Several studies have noted a role for AMPK in mediating resistance to anoikis upon matrix detachment [120,215,216]. For example, AMPK activation in some normal human mammary epithelial cells (HMEC) leads to S116 phosphorylation of PEA15 (phosphoprotein enriched in astrocytes 15 kDa/phosphoprotein enriched in diabetes, PEA15/PED) [217]. Phosphorylated PEA15 binds FADD and prevents recruitment of initiator caspases, thus preventing anoikis upon matrix detachment [217]. In MDA-MB-231 breast cancer cells, AMPK is activated by matrix detachment in a manner that requires LKB1 and CAMKKβ [218]. Under these conditions, no changes were observed in ATP levels upon detachment, and AMPK activation did not depend on detachment-induced changes to FAK or Src, key mediators of signaling that control anoikis [213]. Instead, detachment elicited a spike in intracellular Ca^2+^, which triggered AMPK activation in a manner that required ROS production, and was required for formation of tumour microspheres [218]. Hence, control of anoikis by AMPK may regulate integrin-derived signals in complex ways, but does not appear to control the most integrin-proximal signals such as FAK or Src.

### 3.8. Metabolic Control of Integrins via Alterations in Tumour Microenvironment

The microenvironment of some tumours is acidic (reviewed in [89,219]). The reduced pH of the tumour microenvironment is in part due to the aerobic glycolysis leading to high rates of lactate production, as well as carbonic anhydrase that converts the high rate of CO_2_ produced by the pentose phosphate pathway to H^+^ and HCO_3_^−^. This production is coupled to export of H^+^ and lactate by several transporters, including monocarboxylase transporters (MCTs), Na^+^/H^+^ exchanger (NHE) or the H^+^-ATPase [89].

The change in extracellular pH that results from the altered metabolism of tumours impacts integrin function. Human melanoma (MV3) cells exhibit adhesion and migration that depends integrin α2β1, and either an increase (pH = 7.5) or decrease (pH = 6.6) in extracellular pH impaired cell migration [220]. The impaired migration at low pH resulted from an increase in α2β1-dependent cell adhesion. Molecular dynamic simulations (MDS) revealed that acidic extracellular pH increased activation of αvβ3 integrins [221]. These findings from MDS studies were supported by detection of increased integrin activation at lower pH by flow cytometry and atomic force microscopy-based measurements of αvβ3 engagement of RGD-peptide substrates. The acidic microenvironment of tumours also enhances the activity of some matrix metalloproteases to promote tumour cell invasion, although there is a limit to this, as excessive acidification of the tumour microenvironment may instead impair tumour invasion [222].

## 4. Regulation of Metabolism and Metabolically-Regulated Signals by Integrins

**Figure 2:**
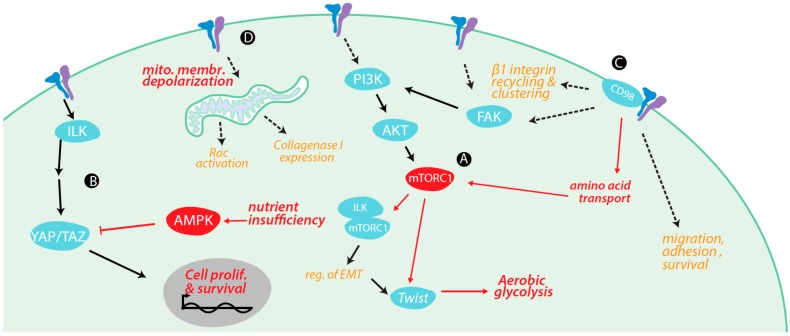
Regulation of metabolism and metabolically-regulated signals by integrins. (**A**) Focal adhesion kinase (FAK) plays a role in the activation of the PI3K-AKT-mTORC1 pathway upon integrin activation. mTORC1 activation by β1 integrin regulates Twist, which promotes EMT and transition to aerobic glycolysis. TGFβ1 stimulation also leads to integrin-linked kinase (ILK)-dependent PI3K-AKT-mTORC1 pathway, as ILK binds to Rictor, a protein component of mTORC1. The ILK-mTORC1 interaction regulates epithelial-mesenchymal transition (EMT); (**B**) integrin signaling regulates the Hippo signaling pathway through ILK. ILK inhibits upstream regulators of YAP/TAZ, which allows for YAP/TAZ activation and translocation to the nucleus, and thus upregulation of genes promoting cell proliferation and survival; (**C**) β1 integrin interacts with CD98, a protein involved in amino acid transport; increased amino acid levels activate mTORC1. CD98 controls β1 integrin recycling and clustering, leading to increased cell surface levels of β1 integrin, and FAK/PI3K signaling events; (**D**) α5 integrin controls Rac activation and collagenase I expression through a signaling mechanism involving mitochondrial depolarization and ROS production, indicating integrin-dependent control of mitochondrial metabolism. Red lines indicated positive regulation (arrowheads) or negative regulation (bars).

Integrins exert control over cell metabolism by a number of mechanisms (Figure 2), including as a result of the activation or potentiation of specific signaling pathways, and also by physical association with cell surface transporters resulting in control of metabolite transport, which we examine here.

### 4.1. Integrin Signaling Cross-Talk with Metabolic Signaling

In general, the signaling pathways activated by integrins upon ECM ligand binding are mitogenic. In particular, integrin-dependent activation of PI3K-Akt-mTORC1 leads to up-regulation of many of the metabolic outcomes of mTORC1 discussed earlier. FAK is a critical mediator of integrin signaling for activation of mTORC1, as evinced by the requirement for FAK in ECM ligation-induced β1-integrin-dependent activation of PI3K-Akt- signaling [18]. ILK also contributes to activation of the metabolically sensitive PI3K-Akt-mTORC1 pathway [223]. In addition, integrins and adhesion regulate the induction of autophagy, such as by engagement of PI3K-Akt and MAPK signaling upon ECM binding, thus promoting cell survival during nutrient or serum deprivation [224].

TGF-β1 stimulation elicits EMT by engaging nuclear translocation of Snail and Snug in a number of cells, including the normal mammary epithelial cell line NMe in a manner that requires ILK [225]. Importantly, TGF-β1 stimulation resulted in formation of a complex containing ILK and Rictor (a protein component of mTORC2). Consistent with ILK exerting control over cell differentiation programming, ILK perturbation in the breast cancer cell line MDA-MB-231 resulted in impaired cell migration and reduced expression of mesenchymal markers such as α-smooth muscle actin. This suggests that ILK exerts control over the engagement of EMT. Moreover, EMT requires substantial reprogramming of genes involved in synthesis and metabolism of many molecules including lipids, nucleotides and amino acids [226]. For example, EMT requires enhanced expression of dihydropyrimidine dehydrogenase and thus enhanced production of dihydropyrimidines from pyrimidines [226]. Hence, by controlling the engagement of EMT, integrins and ILK contribute to the overall alterations in cell metabolism that occur during this differentiation program.

Integrin signaling also controls the Hippo signaling pathway, a critical nutrient sensing system that involves the transcriptional co-activators yes-associated protein (YAP) and transcriptional coactivator with PDZ-binding motif (WWTR1, also known as TAZ), powerful pro-oncogenic regulators [227,228,229]. The phosphorylation of YAP/TAZ by LATS1/2 displaces their interaction with TEAD transcription factors, and phosphorylated YAP/TAZ is further sequestered by binding to 14-3-3 or targeted for ubiquitinylation-dependent degradation. Importantly, this pathway is highly sensitive to metabolic cues in several ways (reviewed by [229]): (i) YAP/TAZ is controlled by Rho signaling, which is in turn sensitive to prenylation dependent on output from HMG-CoA-reductase within the mevanolate biosynthetic pathway; (ii) the TEAD transcription factors are sensitive to glucose metabolism; (iii) YAP/TAZ may be regulated by direct binding to and/or phosphorylation by AMPK, resulting in YAP/TAZ inactivation; and (iv) YAP/TAZ may be negatively regulated by mTORC1 signaling.

Importantly, ILK is a key regulator of signaling through the nutrient-sensitive Hippo pathway, in breast, prostate and colon cancer cells [230]. ILK contributes to the phosphorylation and inactivation of the phosphatase Myosin Phosphatase Target Subunit 1 (MYPT1), resulting in inactivation by enhanced phosphorylation of the MYPT1 substrate Merlin, an upstream regulator of LATS1/2 in the Hippo pathway. In this manner, ILK signaling leads to activation of YAP/TAZ and upregulation of gene transcription for cell proliferation and survival [230]. This work provides a mechanism for integrating metabolic and nutrient sensing cues of the Hippo pathway with cell adhesion and integrin signaling to modulate cell proliferation and survival. Consistent with this work, signaling by fibronectin engagement of α5β1 integrin, but not by laminin engagement of α2β1 integrin, results in inactivation of Merlin, which in turn lead to activation of mTORC1, increased CAP-dependent translation and cell cycle progression [231]. Hence, integrin-derived signals modulate the activity of Merlin, which in turn exerts broad control over several signals including mTORC1 and YAP/TAZ. This integrin-dependent signaling cross-talk allows integration of extracellular and metabolic cues to control cell physiology.

### 4.2. Integrin Signaling Controls Metabolic Pathways

There are several lines of evidence that in addition to interfacing with metabolically sensitive signaling pathways, integrins and integrin-derived signals impact metabolic flux through specific pathways. Antibody engagement of α5-integrin resulted in Rac activation and induction of collagenase I expression in fibroblasts, a phenomenon which required mitochondrially derived ROS production and concomitant integrin-dependent mitochondrial membrane depolarization [232]. Consistent with the regulation of metabolic capabilities by integrin-derived signals, the metabolic reprogramming of cancer cells to increase glycolysis and biosynthetic production is controlled by inputs from integrin signaling. Overexpression of Twist in MCF10 breast cancer cells induces a shift to aerobic glycolysis, and this transition requires β1-integrin signaling to activate the FAK-PI3K-Akt-mTOR signaling axis [233]. These studies suggest that signaling pathways downstream of ligand engagement by integrins alter either mitochondrial function or other metabolic processes in certain contexts, although the mechanism by which this occurs and the consequences on cellular metabolism and energy production remain to be deciphered.

### 4.3. Integrin Control of Nutrient and Metabolite Transporters

β1-integrin interacts with CD98 [234,235,236,237], a dimeric protein comprised of an integrin-binding heavy chain (hc) and one of six known light chains, many of which mediate amino acid transport [238]. Some of the light chains include LAT-1 and LAT-2, which mediate transport of leucine, isoleucine and arginine in exchange for the export of glutamine [239]. In addition to controlling amino acid metabolism and substrate availability for metabolic pathways that in turn control endpoints such as glycan diversity, these amino acids are also critical for activation of mTORC1 signaling [239].

Indeed, CD98 controls integrin signaling, as cross-linking CD98 increases cell surface levels of β1-integrin, integrin clustering and downstream FAK/PI3K signaling [240], and CD98hc overexpression increases cell growth, FAK/PI3K signaling, in a manner dependent on an intact β1-integrin-interaction domain on CD98hc [237,241]. Furthermore, CD98hc contributes to integrin-dependent cell spreading, cell migration, and protection from apoptosis, as evinced by disruption of these phenomena upon genetic deletion of CD98hc in 3T3-L1 adipocyte [242]. While it is tempting to hypothesize that the interaction of β1-integrin with CD98hc provides a physical and functional link to coordinate cell adhesion and regulation of amino acid transport, the expression of a CD98hc molecule unable to bind to CD98 light chains (and thus link to amino acid transport) was similar to wild-type CD98hc in control of cell spreading, cell migration, and protection from apoptosis [242]. Hence, how the physical association between CD98 and β1-integrin may control amino acid transport and metabolism, and how this coordination may contribute to context-dependent cancer growth, survival and metastasis are intriguing questions that remain to be answered.

β1-Integrin interacts with the monocarboxylase transporter 4 (MCT4), which functions as a lactate transporter [243]. In both ARPE-19 and MDCK epithelial cells, β1-integrin co-immunoprecipitates with MCT4 but not the related MCT1, β1-integrin and MCT4 were both present at the leading edge of migrating cells, and perturbation of MCT4 slowed cell migration [243]. Lactate transporters have important functions in cancer cells exhibiting aerobic glycolysis [244]. Taken together with the regulation of integrin activation or ECM association by extracellular pH discussed above, the association of MCT4 with β1-integrin may thus reflect a coordination of metabolically derived proton sources to effect extracellular pH-mediated regulation of integrin function.

Integrins may also control glucose uptake and metabolism, evinced by the observation that detachment from matrix of MCF-10A breast cancer cells leads to a reduction in glucose uptake, reduced ATP levels, increased generation of reactive oxygen species and reduced fatty acid oxidation [245]. The mechanism by which matrix engagement by integrins controls glucose uptake remains to be fully elucidated. However, the rate of glucose uptake and other metabolic properties could be rescued in matrix-detached cells by various methods that increase PI3K-Akt signaling, suggesting that glucose metabolism is regulated by PI3K-Akt signaling, activated by ECM-bound, active integrins.

## 5. Conclusions

The reciprocal regulation of integrin-dependent functions and cell metabolism is an emerging paradigm that effects control over tumours, including by control of cancer cell growth, survival and metastasis. Metabolic sensors such as AMP-activated protein kinase (AMPK), mTORC1 and hypoxia inducible factor (HIF1) integrate metabolic cues to regulate integrin function on many levels, including regulation of transcription, membrane traffic and degradation. Moreover, metabolic flux through specific pathways directly remodels integrin function, such as by control of integrin glycan profile or by control of integrin structure and function by extracellular pH. In turn, integrins and integrin-derived signals control metabolic pathways, either through engagement of specific signaling pathways or by direct association with metabolic enzymes such as membrane transporters.

The reciprocal regulation of metabolism and integrin function has some important implications for the treatment of cancer. Many chemotherapies impose metabolic stress on cancer cells, either by targeting specific metabolic pathways or by inducing cell stress and damage. For example, some of the first chemotherapies, which are still in use to date, target folate metabolism, thus impairing a number of integrated metabolic pathways in cancer cells [246]. While these therapies are effective in many contexts, sub-lethal treatment of cancers with metabolic poisons may induce robust changes in integrin-dependent phenomena such as survival and metastasis. Indeed, treatment of H460 lung cancer cells with sub-lethal cisplatin resulted in increased cell migration, which correlated with increased expression of α4, αv, β1, and β5 integrins [247]. Further, resistance to the mammalian target of rapamycin (mTOR) drug RAD001 in PC3 cells is associated with dramatic up-regulation of β1 and α2 integrins, and reduced adhesion and increased migration and invasion. Importantly, perturbation of α2, α5 or β1 integrins prevented changes in adhesion, migration and chemotaxis in the RAD001-resisant PC3 cells [248]. Indeed, through their interactions with ligands in the tumour microenvironment, integrins have been proposed to have significant roles in the development of tumour resistance to chemotherapies [249,250]. The interwoven control of integrins by cell metabolism and metabolic cues thus make the metabolic control of integrins an important possible mechanism for establishment of cancer resistance to many different chemotherapies. Efforts to resolve how alterations in metabolic signals and flux in cancer cells may establish resistance to specific drugs by control of integrins hold promise for overcoming cancer drug resistance.

An important dimension that has to be considered is the metabolic heterogeneity of cells in healthy tissues as well as in tumours. It is now well appreciated that in addition to inter-tumour differences, there is substantial and significant intra-tumour heterogeneity that strongly impacts tumour properties such as invasiveness and metastasis, as well as response to existing chemotherapies [251,252,253,254,255]. This tumour cell heterogeneity is due in part to the accumulation of mutations within different cells in the tumour and selective pressures within a tumour ecosystem that leads to the establishment of multiple distinct cancer cell populations within the same tumour. For example, glioblastoma multiforme (GBM) can be categorized into subgroups based on expression of specific markers and apparent similarity to various developmental lineages and stages [256,257]. Recent studies have revealed substantial GBM intra-tumour heterogeneity, proposed to arise from a high rate of mutation and cancer cell evolution [258]. Indeed the distinct subgroups of cancer cells within a single tumour exhibited differences in markers for tumour-initiating cells, invasiveness and tumourigenic potential in an animal model [259], indicating the critical importance of understanding the basis and consequences of tumour heterogeneity.

While the importance of intra- and inter-tumour heterogeneity is increasingly becoming apparent, understanding the contribution of metabolic differences between cells to the establishment and outcomes of tumour heterogeneity is also critical. Single-cell metabolomic approaches and other strategies have revealed significant variability in the activity of specific metabolic pathways between cells in tissues and in culture [260]. Consistently, a largely genetically uniform cell population exhibited heterogeneity in the activation of the metabolic stress sensor AMPK upon glucose withdrawal [261]. In tumours, the heterogeneity of the tumour microenvironment with respect to oxygen and nutrient availability adds to intrinsic (e.g., genetic) differences in cell metabolism [262,263]. Thus, when probing the reciprocal regulation of integrin function by metabolism in cancer, it is important to consider the metabolic heterogeneity of cells within tumours.

In summary, the functions of integrins to control cell adhesion, survival, proliferation and migration are interwoven in a network of interdependent regulatory pathways with cell metabolism, which highlights the emerging control of cell physiology by metabolic cues. Importantly, a better understanding of the reciprocal regulation of integrins and metabolism may provide new avenues for the development of biomarkers to improve drug treatment regimes or identify novel drug targets to treat cancer.

## Abbreviations

4EBP1	Eukaryotic translation initiation factor 4E-binding protein 1
AMPK	AMP-activated protein kinase
ARRDC3	Arrestin Domain Containing 3
CMP-sialic acid	Cytidine-5′-monophospho-*N*-acetylneuraminic acid
CPT1C	Carnitine palmitoyltransferase 1C
DOAJ	Directory of open access journals
ECM	Extracellular matrix
EDI3	Endometrial carcinoma differential 3
EGFR	Epidermal growth factor receptor
EMT	Epithelial-mesenchymal transition
FAK	Focal adhesion kinase
FA	Focal adhesion
FADD	Fas-Associated protein with Death Domain
GAP	GTPase Activating Protein
GBM	Glioblastoma multiforme
GEF	Guanyl Exchange Factor
GLUT1	Facilitative glucose transporter 1
GnT-V	β1-6-*N*-Acetylglucosaminyltransferase V
HIF	Hypoxia Inducible Factor
HMEC	Human mammary epithelial cells
ILK	Integrin linked kinase
JNK	c-Jun N-terminal kinase
LAT1/2	L-Type Amino Acid Transporter 1/2
LKB1	Liver Kinase B1
MAPK	Mitogen-activated protein kinase
MCT	Monocarboxylate transporter
MDPI	Multidisciplinary Digital Publishing Institute
mTOR	Mammalian Target of Rapamycin
MDPI	Multidisciplinary Digital Publishing Institute
MYPT1	Myosin Phosphatase Target Subunit 1
*O*-GlcNAc	*O*-Linked β *N*-acetylglucosamine
OGT	*O*-GlcNAc Transferase
PFK	Phosphofructokinase
PI3K	Phosphatidylinotiol-3-kinase
PIP3	Phosphatidylinositol-3,4,5-trisphosphate
PK	Phosphofructokinase
PTEN	Phosphatase and tensin homolog
RGD	Arginyl-glycyl-aspartic acid (motif)
ROS	Reactive oxygen species
RHEB	Ras homologue enriched in brain
TAZ	Transcriptional coactivator with PDZ-binding motif, also known as WWTR1
TSC	Tubular sclerosis complex
SREBP	Sterol regulatory element-binding transcription factor
STGal6 I	β-Galactoside α2,6-sialyltransferase I
UDP-GlcNAc	Uridine 5′-diphospho-*N*-acetylglucosamine
YAP	Yes-associated protein
